# Profiling of Amatoxins and Phallotoxins in the Genus *Lepiota* by Liquid Chromatography Combined with UV Absorbance and Mass Spectrometry

**DOI:** 10.3390/toxins6082336

**Published:** 2014-08-05

**Authors:** R. Michael Sgambelluri, Sara Epis, Davide Sassera, Hong Luo, Evan R. Angelos, Jonathan D. Walton

**Affiliations:** 1 Department of Energy Plant Research Laboratory, Michigan State University, East Lansing, MI 48824, USA; E-Mails: sgambel1@msu.edu (R.M.S.); hongluo@msu.edu (H.L.); angelos1@msu.edu (E.R.A.); 2 Department of Biochemistry and Molecular Biology, Michigan State University, East Lansing, MI 48824, USA; 3 Dipartimento di Scienze Veterinarie e Sanità Pubblica, Università degli Studi di Milano, 10-20133 Milano, Italy; E-Mails: sara.epis@guest.unimi.it (S.E.); davide.sassera@unimi.it (D.S.)

**Keywords:** *amanita*, *lepiota*, amanitin, phalloidin, phallacidin

## Abstract

Species in the mushroom genus *Lepiota* can cause fatal mushroom poisonings due to their content of amatoxins such as α-amanitin. Previous studies of the toxin composition of poisonous *Lepiota* species relied on analytical methods of low sensitivity or resolution. Using liquid chromatography coupled to UV absorbance and mass spectrometry, we analyzed the spectrum of peptide toxins present in six Italian species of *Lepiota*, including multiple samples of three of them collected in different locations. Field taxonomic identifications were confirmed by sequencing of the internal transcribed spacer (ITS) regions. For comparison, we also analyzed specimens of *Amanita phalloides* from Italy and California, a specimen of *A. virosa* from Italy, and a laboratory-grown sample of *Galerina marginata*. α-Amanitin, β-amanitin, amanin, and amaninamide were detected in all samples of *L. brunneoincarnata*, and α-amanitin and γ-amanitin were detected in all samples of *L. josserandii.* Phallotoxins were not detected in either species. No amatoxins or phallotoxins were detected in *L. clypeolaria*, *L. cristata*, *L. echinacea*, or *L. magnispora*. The Italian and California isolates of *A. phalloides* had similar profiles of amatoxins and phallotoxins, although the California isolate contained more β-amanitin relative to α-amanitin. Amaninamide was detected only in *A. virosa*.

## 1. Introduction

The amatoxins, such as α-amanitin, are a group of bicyclic octapeptides produced by some species of mushrooms (phylum Basidiomycota, class Agaricomycetes, order Agaricales). They account for the majority of fatal mushroom poisonings throughout the world. Factors that contribute to their toxicity include resistance to heat and the digestive tract, active intestinal and cellular uptake, and inhibition of RNA polymerase II [1,2]. Symptoms include fulminant hepatic insufficiency; in severe cases liver transplantation is the sole recourse [1].

**Figure 1:**
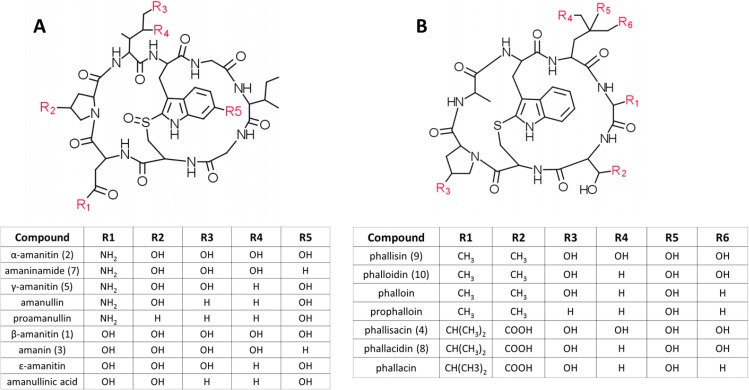
Structures of the (**A**) amatoxin and (**B**) phallotoxin families of bicyclic peptide toxins in mushrooms. Numbers in parentheses after the compound names refer to the peak numbers in the HPLC traces (Figure 3).

Structurally, the amatoxins comprise the amino acid sequences Ile-Trp-Gly-Ile-Gly-Cys-Asn-Pro (α-amanitin) or Ile-Trp-Gly-Ile-Gly-Cys-Asp-Pro (β-amanitin), cyclized by head-to-tail peptide bonds and also a cross-bridge between the Trp and Cys residues. Further diversity among the amatoxins arises from differences in hydroxylations of the side chains, which include 4-hydroxyPro, γ,δ-dihydroxyIle, and 6-hydroxyTrp (Figure 1A).

The phallotoxins, such as phalloidin and phallacidin, are a related class of bicyclic heptapeptides that also contain a Trp-Cys cross-bridge. The core sequences of phalloidin and phallacidin are Ala-Trp-Leu-Ala-D-Thr-Cys-Pro and Ala-Trp-Leu-Val-D-Asp-Cys-Pro, respectively. Differences in the hydroxylations also generate structural diversity among the phallotoxins (Figure 1B). Phallotoxins bind and stabilize F-actin, and their fluorescent conjugates are used as cytological reagents to delineate the actin cytoskeleton. Unlike other known fungal cyclic peptides, the amatoxins and phallotoxins are biosynthesized on ribosomes and are thus classified as ribosomally-synthesized and post-translationally-modifed peptides (RiPPs) [3,4,5].

Although species of *Amanita*, such as *A. phalloides*, *A. verna*, *A. virosa*, *A. ocreata*, and *A. bisporigera*, are the most notorious source of amatoxins and account for most fatal mushroom poisonings, deaths have also been attributed to the ingestion of amatoxin-containing species of *Lepiota*, a genus of small, saprobic mushrooms of worldwide distribution [6,7,8,9,10,11,12]. However, in contrast to *Amanita*, there have been relatively few analyses of the toxic peptide composition of *Lepiota* mushrooms, and to the best of our knowledge none using modern high resolution methods. To date, chemical studies of *Lepiota* species have been restricted to the Meixner test, thin layer chromatography (TLC), and radioimmunoassay (RIA) [8,13,14,15,16]. The Meixner test is not specific, has a high false positive rate, and cannot resolve the different amatoxins [14]. TLC has poor resolution and low sensitivity, and identification relies on nonspecific visualization reagents and comparison of mobilities relative to standards. RIA detects only α- and γ-amanitin and not β-amanitin or phallotoxins [16,17].

In a clinical setting, amatoxin poisoning is often assumed on the basis of severe hepatic malfunction subsequent to mushroom ingestion, even in the absence of chemical evidence [1]. The recent development of molecular methods for the identification of poisonous mushrooms in food or gastric aspirates provides important support for the work of mycologists in clinical cases [18]. However, the development of novel, accurate analytical methods for the toxins themselves is still necessary to confirm amatoxin poisoning and to advance our knowledge of the ecology and biochemistry of these natural products. In order to redress the relative scarcity of information regarding the distribution and abundance of the amatoxins and phallotoxins in the genus *Lepiota*, especially using modern methods of higher sensitivity and resolution, we analyzed six species of *Lepiota* for their toxin content by liquid chromatography-mass spectrometry (LC-MS).

## 2. Results

### 2.1. Mushroom Identification

The species of *Lepiota* and *Amanita* were identified in the field by expert mycologists and the identification were confirmed by DNA sequencing of the ITS regions and comparison to nucleotide sequences in GenBank (Figure S1).

### 2.2. Toxin Analysis

#### 2.2.1. Standards and *A. phalloides*

**Figure 2:**
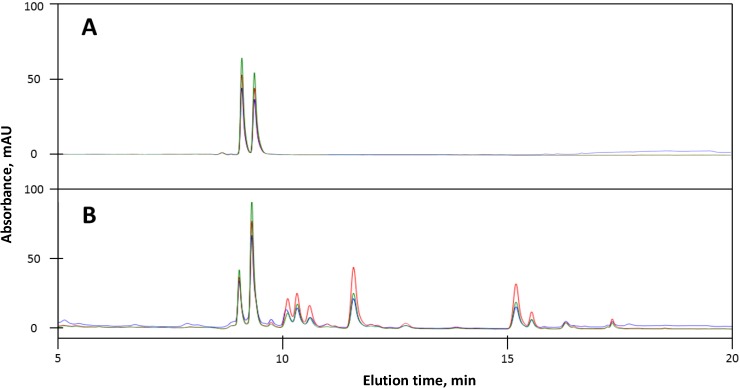
HPLC separation of standards and of an extract of an Italian isolate of *A. phalloides*. (**A**) Mixture of α-amanitin and β-amanitin standards (each 1 μg); (**B**) *A. phalloides* extract. Blue, A_250_; red, A_295_; green, A_305_.

*A. phalloides* is the best-characterized amatoxin and phallotoxin-producing mushroom [19]. This species was included to provide a benchmark for our analysis of *Lepiota* species. It produces multiple forms of amatoxins and phallotoxins including those for which commercially available standards are available, such as α-amanitin, β-amanitin, phalloidin, and phallacidin (Table 1). The UV profile of our samples (Figure 2) is similar to previous studies [20,21,22]. Of particular note is the stronger absorbance at 305 nm compared to 295 nm of compounds that contain the 6-hydroxylated derivative of Trp (e.g., α- and β-amanitin).

**Table 1:** Masses of compounds studied in this paper, and masses observed within each peak of UV absorbance including major adducts (see Figure 3). All masses are monoisotopic from singly charged ions and present at >40% abundance in each spectrum.

Peak Number	Compound	True Mass (Da)	Observed Masses (*m*/*z*)
1	β-amanitin	919.338182	920.3 [M+H^+^], 942.4 [M+Na^+^], 958.4 [M+K^+^]
2	α-amanitin	918.354170	919.3 [M+H^+^], 941.2 [M+Na^+^], 957.2 [M+K^+^]
3	amanin	903.343267	904.3 [M+H^+^], 926.3 [M+Na^+^], 942.2 [M+K^+^]
4	phallisacin	862.316720	863.3 [M+H^+^], 885.3 [M+Na^+^], 901.2 [M+K^+^], 925.3
5	γ-amanitin	902.359252	903.4 [M+H^+^], 925.4 [M+Na^+^], 941.3 [M+K^+^]
6	phallisin II	804.311240	805.3 [M+H^+^], 827.3 [M+Na^+^], 843.2 [M+K^+^], 740.5
7	amaninamide	902.359252	903.3 [M+H^+^], 925.3 [M+Na^+^], 941.2 [M+K^+^]
8	phallacidin	846.321804	847.3 [M+H^+^], 869.3 [M+Na^+^], 885.3 [M+K^+^]
9	phallisin I	804.311240	805.4 [M+H^+^], 827.3 [M+Na^+^], 843.3 [M+K^+^], 864.3, 905.3, 927.1, 942.9
10	phalloidin	788.316330	789.3 [M+H^+^], 811.3 [M+Na^+^], 827.3 [M+K^+^], 848.3
11	unknown	-	889.3 [M+H^+^], 911.3 [M+Na^+^], 927.2 [M+K^+^], 789.2, 811.3, 827.2, 848.3
12	unknown	-	872.5 [M+H^+^], 893.4 [M+Na^+^], 914.5
13	unknown	-	915.4, 937.4, 953.3, 960.6, 974.4
14	unknown	-	755.3, 795.3, 811.2, 832.4, 869.5, 891.5

#### 2.2.2. Lepiota Species

No amatoxins or phallotoxins toxins were observed *in L. clypeolaria*, *L. cristata*, *L. magnispora*, or *L. echinacea* (Supplementary Figures S2 and S3, data not shown). This conclusion was based on UV absorbance and MS analysis using an extracted ion chromatogram (EIC) for all of the known amatoxins and phallotoxins ([M+H^+^] masses). Based on TLC, Gérault and Girre [15] also concluded that *L. clypeolaria*, *L. cristata*, and *L. echinacea* do not produce amatoxins. A conservative detection limit by a combination of UV and mass spectrometry was estimated at 10 ng, compared to 5 μg for TLC [14].

**Figure 3:**
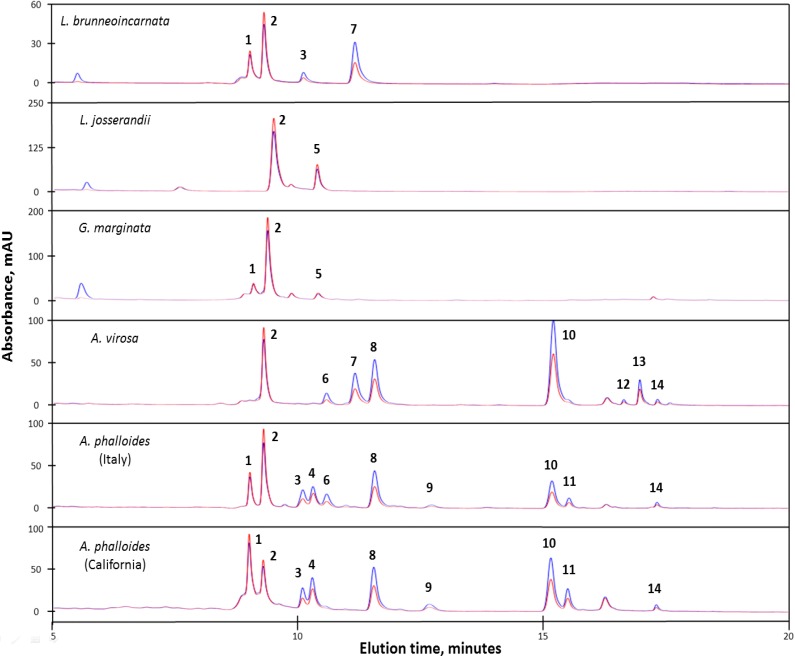
HPLC UV absorption profiles of mushroom extracts from six amatoxin and/or phallotoxin-containing species. Blue, A_295_; red, A_305_. The identities and observed masses for each peak are given in Table 1. Peaks are labeled in order of elution time and shared numbers among extracts indicate the same compound. The shift in retention time of α-amanitin (peak 2) in the *L. josserandii* extract is due to column performance and is within the deviations observed for standards.

*L. josserandii* contained high levels of α-amanitin, as well as a compound with a mass and UV absorbance corresponding to γ-amanitin (peak 5, Figure 3 and Figure S4). α- and γ-Amanitin have the same primary amino acid sequence and therefore could be encoded by the same gene [3]. The absence of β-amanitin in *L. josserandii* is consistent with the TLC results of Haines *et al.* [8], and Beutler and Vergeer [14] reported (also by TLC) the absence of β-amanitin in an American specimen of *L. helveola*. *L. brunneoincarnata* contained α-amanitin, β-amanitin, amanin, and a compound with a mass and UV absorbance corresponding to amaninamide (peak 7, Figure 3 and Figure S5). No phallotoxins were detected in any of the *Lepiota* species (Figure 3 and Figures S2–S6).

Of the mushrooms in this study, *L. josserandi* had the highest level of α-amanitin (3.99–4.39 mg/g dry weight), which is more than three times higher than found in *Amanita* species [8,21,22], including our samples of *A. phalloides* (Table 2).

**Table 2:** α-Amanitin concentrations in mushrooms, calculated from the peak areas of absorbance at 305 nm and a standard curve of α-amanitin. The traces are shown in Figure 3, Figure S4, S5, and S6.

Species	α-Amanitin Content (mg/g dry weight)
*A. phalloides* (Italy)	1.33
*A. phalloides* (USA)	0.88
*A. virosa*	1.39
*G. marginata* (mycelium)	0.57
*L. josserandii* Sample #1	4.24
*L. josserandii* Sample #2	4.39
*L. josserandii* Sample #3	3.99
*L. brunneoincarnata* Sample #1	0.82
*L. brunneoincarnata* Sample #2	0.69

#### 2.2.3. *A. phalloides* from Italy and California

α-Amanitin, β-amanitin, amanin, phallisacin, phallisin, phallacidin, and phalloidin were identified in both isolates of *A. phalloides* (Figure 3). These compounds had the same relative retention times as in other separations using similar chromatographic media and solvents (e.g., reference [21]). Toxins that have been reported in *A. phalloides* at less than 10% abundance relative to α-amanitin (*i.e.*, γ-amanitin, ε-amanitin, amanullinic acid, amanullin, phallacin, and phalloin) were not detected. Possible reasons for this are that our isolates do not make these compounds, some or all of them are extraction artifacts that are not always present, or the sensitivity of our method was inadequate.

Toxin species containing 6-hydroxyTrp absorb more strongly at 305 nm compared to 295 nm [19]. This is illustrated in Figure 2, where α and β-amanitin, both of which contain 6-hydroxyTrp, have stronger absorbances at 305 nm compared to the phallotoxins and amanin, which lack 6-hydroxyTrp and therefore absorb more strongly at 295 nm. In the *A. phalloides* extract, peak 3 (Figure 3) generated an [M+H^+^] ion of 904.3 (*m*/*z*), which could correspond to either ε-amanitin or amanin (Figure 1). However, because peak 3 showed higher absorbance at 295 nm than 305 nm, it probably lacks 6-hydroxyTrp and is thus concluded to be amanin.

Peaks 6 and 9 (Figure 3) both had masses corresponding to that of phallisin ([M+H^+^] = 805.3 *m*/*z*). Using chromatographic conditions similar to ours, Enjalbert *et al.* [21] reported that a standard of phallisin was eluted after phallacidin and before phalloidin, which would correspond to the behavior of peak 9 in our analysis (Figure 3). We therefore conclude that peak 9 is phallisin and peak 6 is an unknown compound. Clarke *et al.* [20] also observed these two peaks of identical mass and named them phallisin I and II, respectively, which nomenclature we retain (Table 1).

*A. phalloides* was introduced into California from Europe not long prior to 1938 [23]. Consistent with this recent immigration, the toxin profiles of the Italian and California isolates were very similar (Figure 3). One difference was in the relative amounts of α- and β-amanitin, the U.S. isolate having higher relative levels of the latter (Figure 3). This difference was also observed by Yocum and Simons [24]. A second difference was that peak 6, the unknown compound with a mass identical to phallisin (named phallisin II), was detected in the Italian isolate but not the American one (Figure 3).

Levels of α-amanitin in the two *A. phalloides* specimens were estimated at 1.33 mg/g dry weight (Italy) and 0.88 mg/gm dry weight (California) (Table 2), comparable to previously reported values (0.75–2.3 mg/g dry weight) [21,22].

#### 2.2.4. *Amanita virosa* and *Galerina marginata*

Since two of the compounds (amaninamide and γ-amanitin) detected in the two toxin-containing *Lepiota* species were not detected in *A. phalloides*, we also analyzed specimens of *A. virosa* and *G. marginata*, which were reported to contain amaninamide and γ-amanitin, respectively [25,26]. A compound with the mass of amaninamide (peak 7, Figure 3) was identified in the *A. virosa* extract with the same retention time and UV absorbance (*i.e.*, higher at 295 than 305 nm) as the compound from *L. brunneoincarnata*. *A. virosa* also contained α-amanitin, phallacidin, and phalloidin. The phallisin analogue (phallisin II) was also present (peak 6, Figure 3). No β-amanitin was observed in *A. virosa*, consistent with some earlier results [24]. However, Ahmed *et al.* [27] reported levels of β-amanitin comparable to α-amanitin in a specimen of *A. virosa* from Japan. *A. virosa* contains a third class of peptides, termed the virotoxins, which are related to the phallotoxins [28], but we observed no masses corresponding to virotoxins in our sample. In the *G. marginata* extract, γ-amanitin, a structural isomer of amaninamide, was identified with the same retention time and UV absorbance (*i.e.*, higher at 305 nm than 295 nm) as the putative γ-amanitin from *L. josserandii*.

In lacking phallotoxins, *G. marginata* resembles the toxin-producing species of *Lepiota* (Figure 3). The genome of *G. marginata* does not contain any phallotoxin-encoding genes [4].

#### 2.2.5. Unidentified Compounds

In addition to the apparent phallisin analogue (phallisin II, peak 6), the observed masses of peaks 11 through 14 (Figure 3), found in *A. virosa* and/or *A. phalloides*, did not correspond to masses of any of the known amatoxins or phallotoxins, although they eluted within the same time frame as the other toxins and absorbed at 295 nm. Unknowns with the same masses as peaks 11 and 12 (889.3 and 872.5 *m*/*z*, respectively) were also found in *A. phalloides* [20].

## 3. Discussion

We report here a high-resolution analysis of the cyclic peptide toxins of *Lepiota* species. Our results are generally consistent with previous reports based on lower resolution or less complete analyses by TLC, RIA, and the Meixner test, and with clinical symptoms from consuming these mushrooms. Our structural identifications were based on a combination of retention time (compared to standards, when available), comparison to extracts of *A. phalloides*, UV absorbance (including diagnostic differences in absorbance at 250, 295, and 305 nm), and mass, and are thus considered to have high reliability. No phallotoxins were detected in any species of *Lepiota*, an observation that to the best of our knowledge has not been previously established. 

In light of recent studies showing that the amatoxins and phallotoxins are synthesized on ribosomes [3,4], some comments on possible biosynthetic relationships of the different toxins and their sub-forms are warranted. In particular, it is probable that the various hydroxylations occur post-translationally, and therefore the less hydroxylated forms (e.g., γ-amanitin, ε-amanitin, amanin, and amaninamide) are intermediates to the more hydroxylated forms (α- and β-amanitin). The order of hydroxylation is apparently not fixed, because hydroxylation of Trp can occur either before or after hydroxylation of Pro and Ile in the amatoxins (Figure 1). Since amanin and amaninamide (and all of the other less hydroxylated forms) were not present in all of the extracts that contained α-amanitin and/or β-amanitin, we conclude that these forms are not artifacts, but rather reflect a true metabolic profile of the compounds in each mushroom.

Another biogenic prediction is that *L. brunneoincarnata* but not *L. josserandi* has a gene for β-amanitin. In species of *Amanita*, a gene encoding the core sequence of α-amanitin (IWGIGCNP) was found in *A. bisporigera* and a gene encoding β-amanitin (IWGIGCDP) was found in *A. phalloides* [3], indicating that the difference between the two amanitins is possibly encoded genetically. (Because the genomes of these two fungi are incomplete, we can only predict, based on their toxin profiles, that *A. bisporigera* has a gene for β-amanitin and *A. phalloides* has a gene for α-amanitin). In contrast, our sample of *L. josserandi* lacked β-amanitin and amanin, both of which are derived from the core sequence IWGIGCDP (Figure 3). This suggests that *L. josserandi* lacks a gene for β-amanitin. On the other hand, β-amanitin was found in *G. marginata* (Figure 3), albeit at much lower levels than α-amanitin, even though no gene encoding β-amanitin is present in its complete genome [4]. Therefore, it cannot be excluded that some toxin-producing fungi might contain an enzyme, such as a deaminase, that can convert the Asn in amatoxins to Asp.

Our results show that *L. josserandi* and *L. brunneoincarnata* produce amatoxins, and that *L. clypeolaria*, *L. cristata*, *L. echinacea*, and *L. magnispora* do not. Species of *Amanita* (such as *A. muscaria*) and *Galerina* (such as *G. hybrida*) that do not produce amatoxins and/or phallotoxins lack the genes encoding the core toxin sequences [3,4]. If these precedents hold true for the genus *Lepiota*, then toxin nonproducing isolates of *Lepiota* are predicted to lack the genes for amatoxins, and all species of *Lepiota* are predicted to lack genes encoding phallotoxins. 

Despite our results, the species in which no amatoxins were found should not be considered edible. Species of *Lepiota* can be difficult to identify without molecular tools, and hybridization between toxin-producing and nonproducing species is not implausible.

## 4. Experimental Section

### 4.1. Biological Material

All *Lepiota* species and the Italian specimens of *Amanita* (*A. phalloides* and *A. virosa*) were collected in the Lombardy region of Italy during the period May through November, 2012 or 2013. The three samples of *L. cristata* were collected in different locations within Cologno Monzese and Rozzano (both in the province of Milan). The three samples of *L. josserandi* were collected in different locations in Cologno Monzese and Cassina de Pecchi (province of Milan). Mushrooms were morphologically identified by local expert mycologists with standard taxonomic keys [29,30,31]. The specimens were freeze-dried or dried at room temperature and then stored at −80 °C. *L. josserandii* is now considered to be a synonym for *L. subincarnata* J.E. Lange [32,33].

*A. phalloides* was collected in Alameda County, California, in December, 2012. Samples were freeze-dried and stored at −80 °C before analysis. A monokaryotic isolate of *G. marginata* was obtained from Centraalbureau voor Schimmelcultures (CBS), Utrecht, Netherlands (catalog number 339.88) and grown as described [4].

### 4.2. ITS Sequencing

The internal transcribed spacer (ITS) regions of the *Lepiota* species were amplified using primer pairs ITS1 and ITS4 [34]. For template preparation, ~1 mg of dried mushroom was homogenized with a tissue grinder in 50 μL of lysis buffer as described [35]. The samples were centrifuged at 15,000× *g* in a microfuge (Eppendorf 5415D) for 2 min and 1 μL of the supernatant used as the PCR template. PCR was performed under standard conditions using RedTaq polymerase (Sigma, St. Louis, MO, USA) in a total reaction volume of 20 μL. The DNA products of the reaction were cloned into pGEM-T Easy vector (Promega, Madison, WI, USA) and sequenced.

### 4.3. Extraction

The freeze-dried fungal tissues were frozen in liquid nitrogen, ground with a mortar and pestle, and suspended in methanol: H_2_O:0.01 M HCl, 5:4:1, at a concentration of 10 mL/g tissue [21]. Following a 1-h incubation at room temperature, the extracts were centrifuged at 10,000× *g* for 10 min and the supernatants filtered through a 0.22 μm filter (Millex polyvinylidene fluoride, GV4, Thermo Fisher Scientific, Waltham, MA, USA). Samples were stored at −70 °C until analysis. Immediately prior to HPLC fractionation, the extracts were diluted with 20 mM ammonium acetate, pH 5, to a concentration of 20 mg dry weight/mL. Standards of α- and β-amanitin were purchased from Sigma (St. Louis, MO, USA).

### 4.4. Liquid Chromatography-Mass Spectrometry (LC-MS)

A number of methods have been applied to the analysis of the *Amanita* toxins in mushrooms and bodily fluids such as urine, with the goal of facilitating the clinical diagnosis of mushroom poisoning. Most methods rely on some combination of liquid chromatography and mass spectrometry [27,36,37,38].

The fungal extracts were separated on a reversed-phase Proto 300 C18 column (Higgins Analytical; 5 µm, 250 × 4.6 mm) using an Agilent series 1200 HPLC equipped with a multi-wavelength detector. Solvent A was 0.02 M ammonium acetate, pH 5, and solvent B was acetonitrile (HPLC grade, EMD Millipore, Billerica, MA, USA). The program was 10% B for 4 min, then 18% B for 6 min, then a linear gradient from 18% B to 100% B over 20 min, at a flow rate of 1 mL/min. In each run, the equivalent of 0.6 mg tissue was injected in a volume of 30 μL, except *G. marginata*, for which the equivalent of 3 mg was injected. The eluate was monitored at 250, 295, and 305 nm. Mass analysis of the eluate was performed with an Agilent 6120 single-quadrupole mass spectrometer in positive polarity mode using a scan range of 700–1000 *m*/*z*. Ions were generated by electrospray with a capillary voltage setting of 5 kV and a drying gas (nitrogen) temperature of 350 °C and a flow rate of 12.0 L/min.

## 5. Conclusions

Some species of *Lepiota*, which have been known to cause human poisonings, produce α-amanitin and β-amanitin, but not phallotoxins. Levels of α-amanitin in *L. josserandii* are three to four times higher than in *A. phalloides*, *G. marginata*, or *L. brunneoincarnata*.

## Supplementary Files

Supplementary File 1
