# Supplementary material for: Profiling of Amatoxins and Phallotoxins in the Genus Lepiota by Liquid Chromatography Combined with UV Absorbance and Mass Spectrometry
Source: Toxins (Basel). 2014 Aug 5;6(8):2336–47. doi: 10.3390/toxins6082336 (PMC4147585; doi:10.3390/toxins6082336)

Supplementary Data

Table S1. Retention times of compounds 1–14 (see Figure 3).

Peak Number	Compound	*A. phalloides* (Italy)	*A. phalloides* (California)	*A. virosa*	*L. brunneo-incarnata*	*L. josserandii*
1	β-amanitin	9.021	9.003	-	9.005	-
2	α-amanitin	9.300	9.289	9.289	9.290	9.496
3	amanin	10.100	10.092	-	10.095	-
4	phallisacin	10.306	10.290	-	-	-
5	γ-amanitin	-	-	-	-	10.384
6	phallisin-I	10.585	-	10.568	-	-
7	amaninamide	-	-	11.150	11.144	-
8	phallacidin	11.563	11.542	11.549	-	-
9	phallisin-II	12.724	12.674	-	-	-
10	phalloidin	15.176	15.151	15.188	-	-
11	unknown	15.524	15.495	-	-	-
12	unknown	-	-	16.624	-	-
13	unknown	-	-	16.951	-	-
14	unknown	17.315	17.299	17.311	-	-

Figure S1. ITS sequences from the six species of *Lepiota*.

Lepiota brunneoincarnata

TCCGTAGGTGAACCTGCGGAAGGATCATTATTGAATAAACTTGGTGGGTTGTTGCTGGCTT
CTTGGAGCATGTGCACGCTCATCGACTTTATCCATCCACCTGTGCACCTTCTGTAGTCTTC
GAAATGAAAGCGGCTGAGCCTCGATGGGCATTTTGCCCTATCGGATGTGAGGAATGCTTT
TGTGAAGGCATGGCTCTCCTCAAAGGCCTGTGATCGTTTCTTGGACTATGTTTTTCCATAT
ACCACATAGCATGTTGTAGAATGTATCGGTGGGCCTCTGTGCCTATAGAACTCAATACAA
CTTTCAGCAACGGATCTCTTGGCTCTCGCATCGATGAAGAACGCAGCGAAATGCGATAAG
TAATGTGAATTGCAGAATTCAGTGAATCATCGAATCTTTGAACGCACCTTGCGCTCCTTGG
TATTCCGAGGAGCATGCCTGTTTGAGTGTCATTTAATTCTCAACCATGCTGGCTTTGTAAA
GGTCAGTTGTGGCTTGGATTGTGGGGGTATTCCTGCGGGTCTCTCTTGAGGTCGGCTCCCC
TAAAATGCATTAGCAGAACCGTTTGCGGTCAGTCGCAGGTGTGATAATTATCTACGCCAA
AGACCAAGGCTGCTCTCTGTTTGTTCAGCTTCTAATTGTCTCGGGACAAATTTTTTTGAAT
GTTTGACCTCAAATCAGGTAGGACTACCCGCTGAACTTAAGCATATCAATAAGCGGAGGA

L. magnispora (synonym *L. clypeolaria*)

TCCGTAGGTGAACCTGCGGAAGGATCATTATTGAATAACTATGGTGGGTTGTTGCTGGCTT
CTTGAAGCATGTGCACACCTGCTGTCTTTATCTATCCCACTGTGCACCATTTGTAGTCTTG
GAGGGGGAAGAGCGGTGAAGCTCACATGCCCCCCCTTCCGGGTCTATGTCTTTTCCACAA
ACATTGTAGTATGTCACAGAATGTAATCAAAGGGTCTTTGTGCCCATAAAACTATATACA
ACTTTCAGCAACGGATCTCTTGGCTCTCGCATCGATGAAGAACGCAGCGAAATGCGATAA
GTAATGTGAATTGCAGAATTCAGTGAATCATCGAATCTTTGAACGCACCTTGCGCTTCTTG
GTATTCCGAGGAGCATGCCTGTTTGAGTGTCATTAAATTCTCAATCCCTTCCAGTATTCTG
GTTGTGGCTTGGATATTGGGGGTTTCTGCAGGCCTTATTATGTTGAGGTCAGCTCCCCTAA
AATACATTAGCAGAACTGTTTGCGGTCTGTCACTGGTGTGATAATTATCTGCACCAAGGCT
GCTTTCTATCTTGTTCAGCTTCCAACCGTCTTCTTGGAGACAACTATTGAACATTTGACCTC
AAATCAGGTAGGACTACCCGCTGAACTTAAGCATATCAATAAGCGGAGGA

L. echinacea

TCCGTAGGTGAACCTGCGGAAGGATCATTATTGAATAAACCTGGTGGGCTGTAGCTGGCT
CTTCGGAGCATGTGCACRCTCATCCACTTTTATCCATCCACCTGTGCACCATGTGTAGTCT
TGGGGGAGAAAGATTTGCGGTCCCGCTGTgGGCTTGTGAAGACGTCCTCTCAATTCTATGT
TTTTCATATACCACRTAGTATGTTGCAGAATGTAtATAACGGGCCTATGTGCCTATAAAAC
ACAATACAACTTTCAGCAACGGATCTCTTGGCTCTCGCATCGATGAAGAACGCAGCGAAA
TGCGATAAGTAATGTGAATTGCAGAATTCAGTGAATCATCGAATCTTTGAACGCACCTTG
CGCTCCTTGGTATTCCGAGGAGCATGCCTGTTTGAGTGTCATTATATTCTCAACCCTTcCCA
GtTWTaaTgaCtTGGGTaaGTGGATTGGATTGTGGGGGCTTGCTGGTCGCTTTACTGCGGTCGG
CTCCTCTGAAATGTATTAGCGGAACTGTTTGCGGTCcCGTCACTGGTGTGATAATTATCTac
GcCgaAgACgAAGGCTGCTCTCTATACGTTCAGCTTATAATCaGTCCCCTcTGGtGGACAACT
TTTGAAAGTTTGACCTCAAATCAGGTAGGACTACCCGCTGAACTTAAGCATATCAATAAG
CGGAGGA

L. cristata

TCCGTAGGTGAACCTGCGGAAGGATCATTATTGAATAAACTTGGTAGGTTGTAGCTGGCTT
TTCGAAGCATGTGCACGCCTACTATCTTTATCCATCCACCTGTGCACCCTTTGTAGTCTTGG
AGGACAAGAGCGGCTGACTCCTCGAACGGCTTCTTCTAGCCTTTCGGATGTGAGGGATGCT
GTGTGAAAGCACRGCTCTCCTCAATGGCTCGCAATTTCCTCTAGGTCTATGTCTTTTCCATA
TACCACATAGTATGTTGTAGAATGCATTATATGGGCCCATGTGCCTATAAAACTCAATACA
ACTTTCAGCAACGGATCTCTTGGCTCTCGCATCGATGAAGAACGCAGCGAAATGCGATAAG
TAATGTGAATTGCAGAATTCAGTGAATCATCGAATCTTTGAACGCACCTTGCGCTCCTTGGT
ATTCCGAGGAGCATGCCTGTTTGAGTGTCACTAAATTCTCAACCACTCCAGCCTTTGCGGGT
TGGATGTGGCTTGGATGTTGGGGGTTTCTGCGGGCCTCTCTTTTGAGGTCGGCTCCCCTGAA
ATGCATTAGCGGAACCGTTTGCGGTCCGTCGCCGGTGTGATAATTATCTACGCCATAGACG
AAGGCTGCTCTCTGTATGTTCAGCTTCTAACTGTCCCCTGTGGACAACTTTTGAACGTTTG
ACCTCAAATCAGGYAGGACTACCCGCTGAACTTAAGCATATCAATAAGCGGAGGA

L. josserandii (synonym *L. subincarnata*)

TCCGTAGGTGAACCTGCGGAAGGATCATTATTGAATAAACATGGTGGGTTGTCGCTGGCT
CCTTGGAGCATGTGCACGCTCATCGTCTTTATCCATCCACCTGTGCACCTTTTGTAGTCTTG
GGAAATGAATGCAATGGAACCTCGATAGGTTTTTCAGCCTTTCGGATGTGAGGAATGCTT
TGTGAAAGCATGGCTCTTCTCAATAGCCTTGCAATCGTTACTCAGACTATGTTTTTCATAC
ACCATGTAGTATGTTTGCAGAATGTATCAATGGGCCTCTGTGCCTATAAAACTCAATACA
ACTTTCAGCAACGGATCTCTTGGCTCTCGCATCGATGAAGAACGCAGCGAAATGCGATAM
GTAATGTGAATTGCAGAATTCAGTGAATCATCGAATCTTTGAACGCACCTTGCGCTCCTTG
GTATTCCGAGGAGCATGCCTGTTTGAGTGTCATTTAATTCTCAACCACAAAGGCTTTTGCG
AGCTTTTGTGGATTGGACGTGGGGGTAACTGCAGGCCTTCCCAGGTCAGCTCCCCTAAAA
TGCATTAGCGGAACCGTTTGCGGTAACCAGTCGCCAGGTGTGATAATTATCTACGCCAAT
AGACATGAACTGCTCTCTGTTGTTCTGCTTCAAATTGTCTTGCTAGACAACTTTTGAATGTT
TGACCTCAAATCAGGTAGGACTACCCGCTGAACTTAAGCATATCAATAAGCGGAGGA

Figure S2. UV trace of *L. cristata* Sample #2. No amatoxins or phallotoxins were detected by UV or MS.

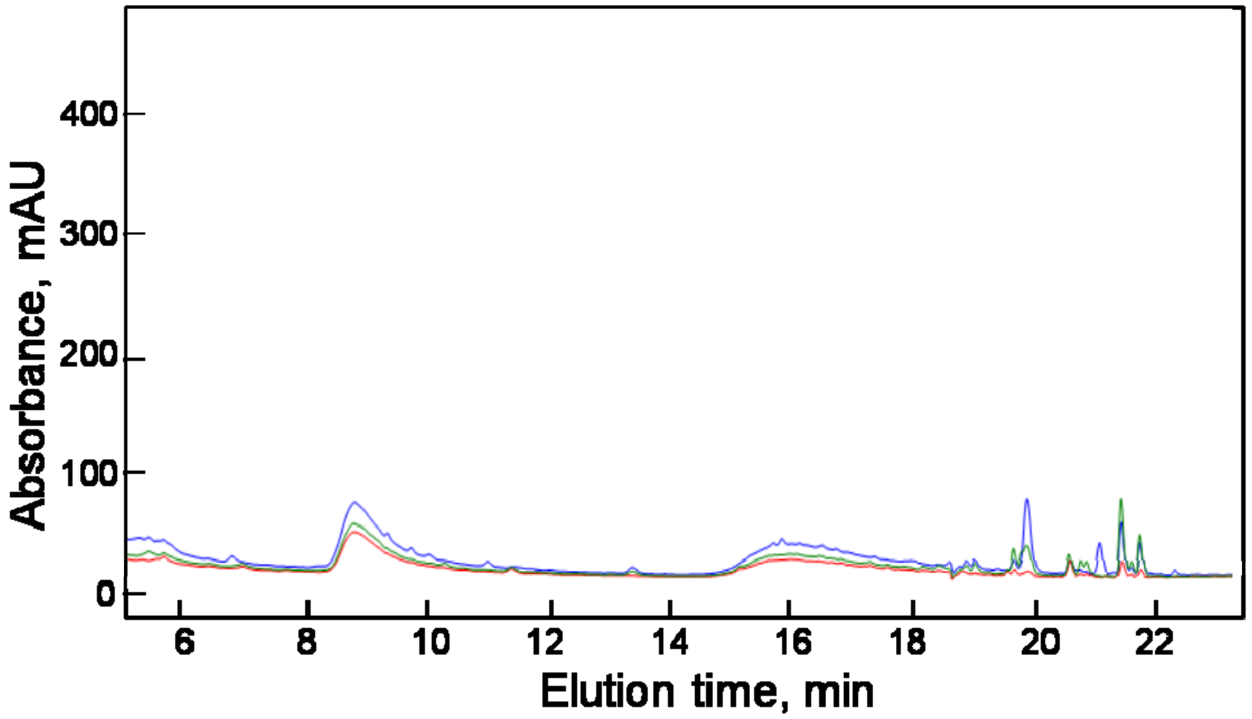

Figure S3. UV trace of *L. cristata* Sample #3. No amatoxins or phallotoxins were detected by UV or MS.

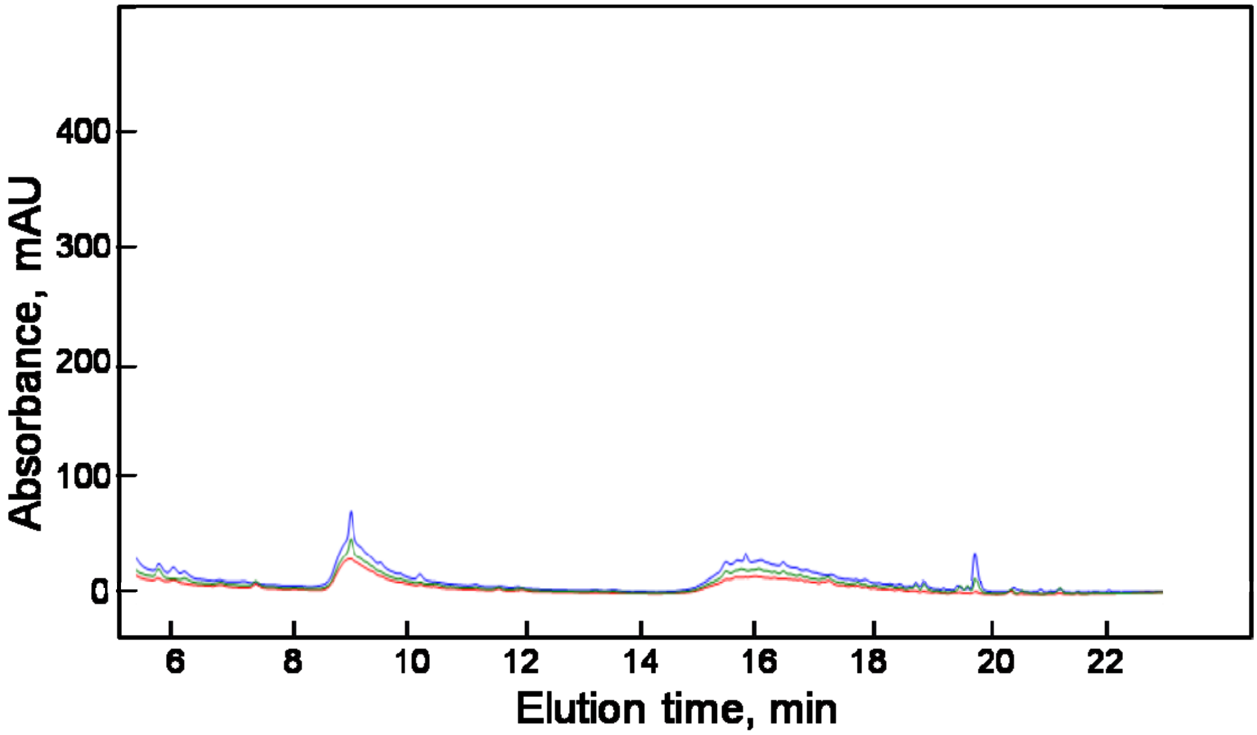

Figure S4. UV trace of *L. josserandi* Sample #2. By UV and MS, α-amanitin, γ-amanitin, and a trace of amaninamide were present.

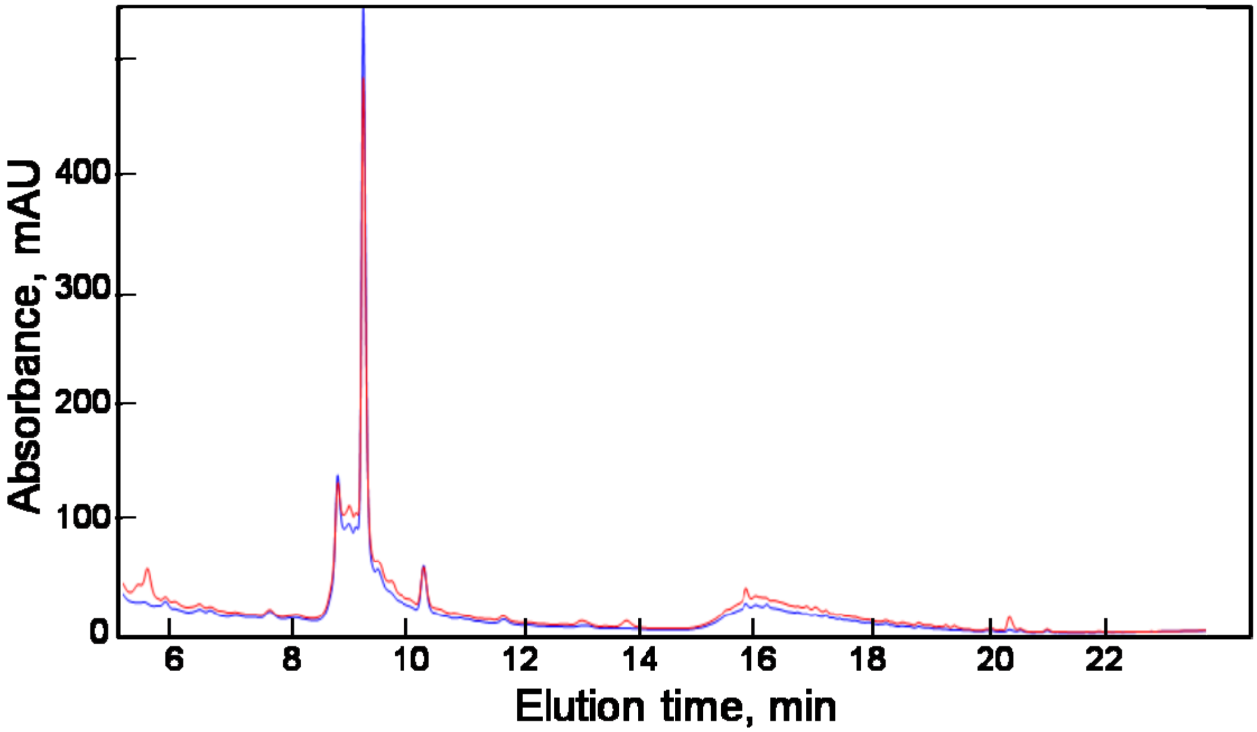

Figure S5. UV trace of *L. brunneoincarnata* Sample #2. By UV and MS, α-amanitin, β-amanitin, and traces of amanin and amaninamide were present.

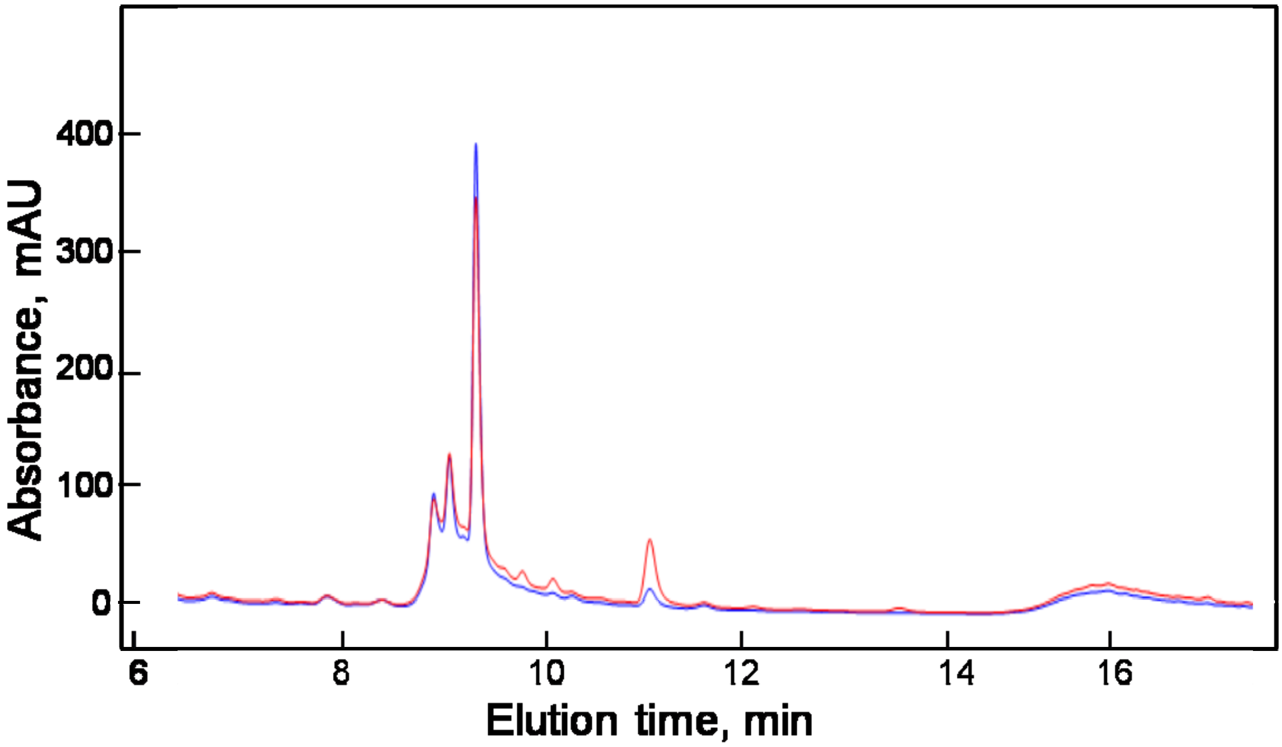

Figure S6. UV trace of *L. josserandi* Sample #3. By UV and MS, α-amanitin and γ-amanitin were present.

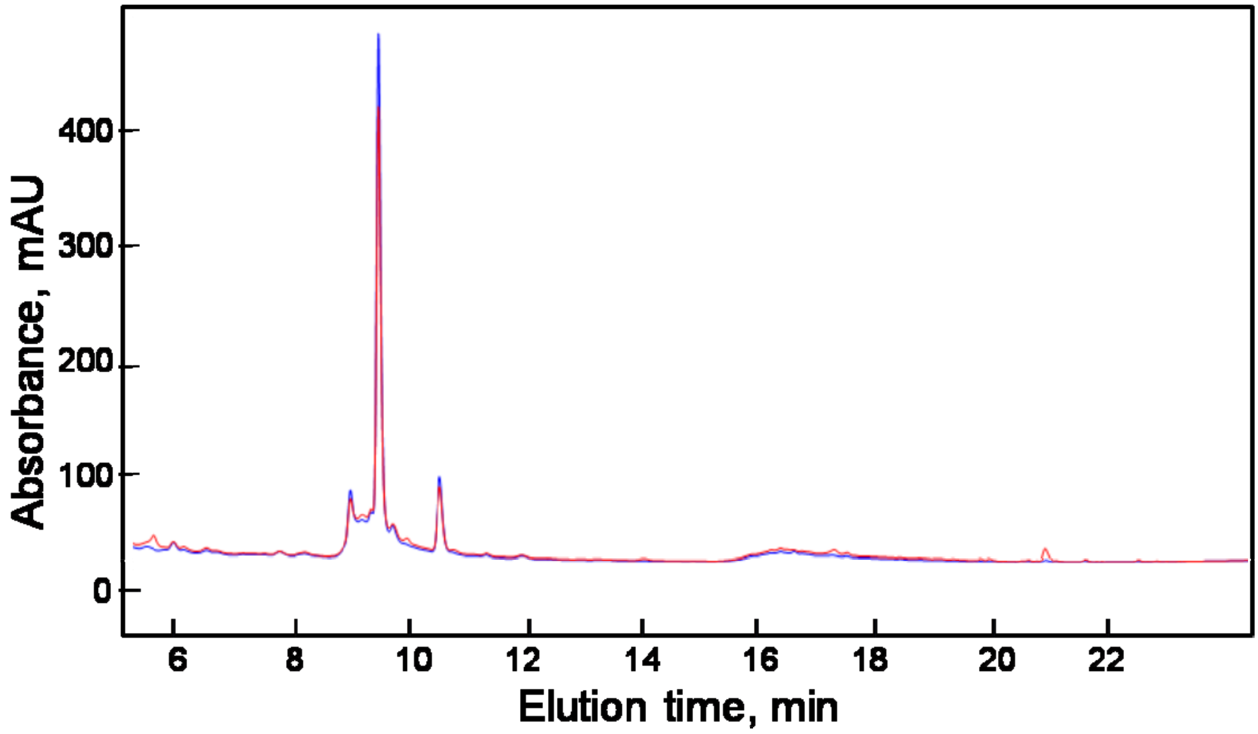

Supplement: Supplementary File 1 [file toxins-06-02336-s001.pdf]
